# Effect of Radial Artery Compression with a Novel Automatic Pressure-Controlled Radial Compression Device: A Short-Term Prospective Interventional Pilot Study

**DOI:** 10.1155/2023/7533702

**Published:** 2023-03-07

**Authors:** HaiZhen Xu, Junya Cheng, DanYing Zhang, Liang Shen, Yingjie Jiang, ChangLin Zhai

**Affiliations:** ^1^Department of Nursing, The First Hospital of Jiaxing (Affiliated Hospital of Jiaxing University), Jiaxing314000, China; ^2^Department of Cardiology, The First Hospital of Jiaxing (Affiliated Hospital of Jiaxing University), Jiaxing314000, China

## Abstract

This study was conducted to design a novel radial compression device with the function of automatic pressure control and evaluate the feasibility and safety of this new technique. Patients who underwent transradial access (TRA) coronary angiography and percutaneous coronary intervention (PCI) in the First Hospital of Jiaxing between August 2021and October 2021 were prospectively enrolled in this pilot interventional study. The patients were grouped in a 1 : 1 ratio to receive compression with a novel device (the experimental group) or a conventional device without pressure control (the control group). The primary endpoint was the compression time, and the main secondary endpoints were rebleeding, upper-limb swelling, radial artery occlusion (RAO), and device-related pressure injury (DPI). Eighty-four patients were enrolled in this study. No significant differences were found in the baseline clinical characteristics between the two groups. Compared with the control group, the compression time in the experimental group was significantly reduced (207.4 ± 15.5 vs. 378.1 ± 19 min, *p* < 0.001). Besides, the rate of upper-limb swelling was also significantly lower in the novel device group (2.4% vs. 85.7%, *p* < 0.001), as well as the rate of DPI (19.05% vs. 100%, *p* = 0.005). Furthermore, the pain score in the experimental group was significantly lower than in the control group (0.79 ± 0.42 vs. 1.83 ± 0.58, *p* < 0.001). There were no significant differences in the rate of rebleeding (7.1% vs. 14.3, *p* = 0.48) between the two groups. In addition, no RAO occurred in any of the groups. The novel automatic pressure-controlled radial compression device could reduce the hemostasis time and decrease the rate of adverse complications. It might be a promising and effective compression device in TRA coronary invasive procedures.

## 1. Introduction

Currently, percutaneous coronary intervention (PCI) is one of the most extensive and effective treatment strategies used for patients with coronary artery disease (CAD) [1]. Transradial access (TRA) has become globally accepted as the preferred vascular site approach for diagnostic or interventional coronary procedures [1, 2]. It is recommended as a Class IA indication in the invasive management of CAD patients undergoing PCI [3]. Compared with transfemoral access (TFA), TRA can significantly reduce the rate of access site complications and major adverse cardiovascular events (MACE), whereas it can improve postprocedural comfort and clinical outcomes, which have been reported in previous studies [4–9]. Moreover, the radial artery is easier to compress due to its superficial placement, and patients undergoing TRA coronary invasive procedures can move immediately after the procedure. Consequently, postoperative nursing can also be alleviated [10, 11].

Nevertheless, despite the advantages of TRA in the perioperative and long-termfollow-up compared with TFA, access site complications, such as radial artery occlusion (RAO), upper-limb swelling, and rebleeding, remain huge challenges [6, 7, 12]. Furthermore, numerous factors have been associated with TRA-related complications, among which compression time or time to hemostasis might be crucial factors. Hence, a radial compression device that considers both hemostasis and compression time might have great clinical application prospects.

**Figure 1:**
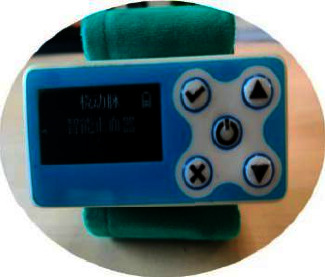
A new radial artery compression device with a pressure control technique.

Herein, we designed a new radial artery compression device with a pressure control technique (Figure 1) that can automatically reduce the pressure exerted on the punctured radial artery and assessed its availability and safety in clinical practice.

## 2. Methods

### 2.1. Study Design and Participants

This prospective, single-center, interventional pilot trial included patients with coronary heart disease requiring coronary artery puncture or angiography treated in the Cardiology Department of a large tertiary grade A hospital in Jiaxing, Zhejiang Province, China. In this trial, we compared two radial artery compression devices after TRA coronary procedures. This study was conducted in accordance with the Helsinki Declaration II, and the prospective trial was approved by the Ethics Committee of the First Hospital of Jiaxing (Ethical label: LS-2020-179).

Eighty-four patients admitted to our hospital between August 2021 and October 2021 for invasive coronary assessment were enrolled. Depending on the type of device used after TRA, the patients were divided into two groups: the novel device group (*n* = 42) and the conventional device group (*n* = 42). The inclusion criteria were (1) patients with coronary heart disease according to diagnosis criteria, (2) patients ≥18 years of age, (3) patients with clear consciousness with a certain level of understanding and expression ability, and (4) patients who are willing to participate in the study and provide written informed consent. The exclusion criteria were (1) patients with upper-limb disability or deformity, (2) patients with local skin or tissue edema or infection, (3) patients with abnormal coagulation function, or (4) patients with severe comorbidities.

### 2.2. Use of the Automatic Pressure-Controlled Radial Compression Device

The automatic pressure-controlled radial compression device is presented in Figure 1. The device contains a panel that shows the time and pressure, along with buttons controlling the pressure value and compression depth. The device's size is about 4 × 2 × 3 cm, with a mechanical spring and rubber gasket connecting the machine and sterile gauze covering the puncture site's surface. In addition, the compression device is surrounded by a self-adhesive elastic bandage.

The detailed operation steps are as follows (as presented in Table 1): after completion of the transradial procedure, the sheath was pulled out to 2 to 3 cm, the automatic compression device was put over the puncture site, the device was switched on, and the pressure was adjusted. When the pressure reached 220 mmHg, the arterial sheath was quickly removed and observed for 3–5 min to ensure no bleeding. After returning to the ward, the pressure was adjusted to 20 mmHg higher than the systolic blood pressure (SBP). After 2 h, the pressure automatically decreased at a uniform velocity (30 mmHg/h). Once the pressure was lower than the SBP with an alarm notification, the device was removed if there was no bleeding at the puncture site. The next day, vascular ultrasound was used to evaluate the radial artery patency.

The pressure value of the compressor was set to 220 mmHg for the first time. When the patient returns to the ward, the pressure setting is started according to the patient's blood pressure value. The reasons for setting it to 220 mm Hg for the first time are as follows: (1) at the end of the operation, according to the SBP of the patient, the operator adjusted the pressure of the hemostat at 150–200 mmHg, with a reference value of 30–50 mmHg higher than the patient's systolic blood pressure, and the maximum pressure was not >250 mmHg. The catheter room nurse adjusts the hemostatic pressure by monitoring the thumb SPO_2_ on the operative side. When SpO_2_is ≥95%, the pressure of the hemostat will not interfere, and the pressure of the hemostat will remain unchanged. (2) When the SBP of a patient exceeds 180 mmHg clinically, except for critically ill patients, the PCI operation would be suspended and restarted once the SBP is <180 mmHg. Therefore, based on the upper limit of 180 mmHg of systolic blood pressure that can be used for PCI, we increased it by 40 mmHg (i.e., the average value of 30–50 mmHg) to obtain 220 mmHg, and the SpO_2_ of the thumb of the operating limb is monitored to remain ≥95% to ensure the blood supply. (3) The research on the appropriate pressure value of the radial artery compressor is inconclusive. The first pressure value of the compressor was mainly determined by experience.

### 2.3. Use of a Conventional Compression Device

According to the color code pressure reference index in the product manual of the hemostat, 150–200 mmHg was generally selected for the first time, that is, the middle or bottom of the green color code. The pressure was increased by 25 mmHg every time the cap was tightened, and the maximum pressure did not exceed the yellow color code (200–250 mmHg). During the operation, the operator confirmed the puncture point of the radial artery. After the operation was completed, the sheath of the artery was withdrawn with a range of 1–2 cm. The puncture point of the patient was ensured and covered with sterile gauze. Then, the pressure plate of the hemostat was firmly pressed vertically on the puncture point, and another operator fixed the adhesive buckle on the patient's wrist, tightened and stuck it around the wrist, rotated the handle of the hemostat clockwise to exert pressure on the puncture site through the pressure plate, and at the same time, pulled out the arterial sheath and adjusted the handle of the hemostat until no blood leakage was seen [13–16]. The handle was turned by one turn every hour for progressive decompression. One turn of the handle is approximately equivalent to 30 mmHg.

### 2.4. Study Endpoint

The primary endpoint was the compression time after transradial procedure completion, which refers to the time from the withdrawal of the sheath to the removal of the compression device with satisfactory hemostasis. The secondary endpoints included (1) rebleeding, defined as any visible bleeding from the access site after initial removal of the compression device, (2) upper-limb swelling on the operative side, (3) RAO, defined as no anterograde flow in the radial artery detected by vascular ultrasound, (4) device-related pressure injury (DPI), and (5) pain score, determined using the NRS Pain Assessment Scale.

### 2.5. Statistical Analysis

The continuous variables with a normal distribution were expressed as means ± standard deviations and analyzed using Student's *t*-test between the two groups. The categorical variables were expressed as *n* (%) and analyzed using the *χ*^2^-test. All statistical analyses were conducted using SPSS 22.0 (IBM Corporation, Armonk, NY, USA). Two-tailed*p* < 0.05 was considered statistically significant.

## 3. Results

### 3.1. General Characteristics of the Participants

Among the 84 enrolled patients, 58 were male, and their average age was 65.30 years. The baseline characteristics are summarized in Table 2. There were no significant differences in the rate of comorbidities between the two groups, as well as the value of activated partial thromboplastin time (APTT), prothrombin time (PT), and the platelet counts (*p*values 0.168, 0.322, 0.306, and 0.802, respectively). Among the 84 participants, only seven underwent PCI, and the dose of heparin in the two groups did not significantly differ (4940.48 ± 938.36 vs. 5285.71 ± 918.26, *p* = 0.092).

### 3.2. Study Endpoints

All patients had the radial artery compression removed after a certain time. Study endpoints are presented in Table 3. The mean compression time in the experimental group was 207.4 ± 15.5 min, which was significantly less than 378.1 ± 19 min in the control group (*p* < 0.001). In both groups, rebleeding occurred after the removal of the compression device occurred in three patients in the experimental group and six in the control group (7.1% vs. 14.3%, *p* = 0.480). The rates of upper-limb swelling and DPI were significantly lower in the novel device group compared with the control group (2.4% vs. 85.7% and 19.05% vs. 100%, *p* = 0.005, respectively). In addition, the pain score in the experimental group was also significantly lower than the control group (0.79 ± 0.42 vs. 1.83 ± 0.58, *p*p < 0.001). Finally, no RAO occurred in any of the groups.

## 4. Discussion

In this study, we evaluated the efficacy of a new radial artery compression device with automatic pressure control and found that compared with the conventional device, this novel device could significantly reduce the compression time without increasing the rate of rebleeding, upper-limb swelling, DPI, and RAO. Moreover, the novel device could alleviate the pain caused by compression and further increase the comfort and satisfaction of the patients [17, 18].

In clinical practice, it is always recommended to compress the radial puncture site for at least 4 h after coronary intervention [19, 20]. In this study, the average compression time in the intervention group was 207 min. Moreover, we found no increased vascular complications such as rebleeding and RAO. Globally, several compression devices exist, such as StatSeal® for TRA, TR Band™ (Terumo, Japan), and Safeguard® (Merit Medical, USA). In our hospital, the most widely used compression device is shown in Figure 1. Our team designed and developed a new device with an automatic decompression function, which can regulate the pressure precisely and replace manual decompression. The traditional compression device cannot adjust the compression intensity according to the dynamic blood pressure as the new compression device in this study can do, and the selection accuracy of the compression point is high. Once the compression point deviates from the radial artery, blood leakage into the skin is not easy to detect early, leading to complications such as hematoma and forearm swelling. Therefore, the average compression time is longer. During the intervention, this study mainly focused on the effectiveness and safety of hemostasis. The literature agrees that the compression time should be kept as short as possible without compromising patient safety [21–24]. The control of compression time using the device is still conservative, but it has been significantly shortened by more than 2 h. Indeed, in the intervention group, the average time was 207 min (about 3.4 h). In the follow-up study, we will continue to explore the advantages of this compression device regarding compression time and the safety and feasibility of shortening the compression time to about 2 h.

Our results revealed a significant reduction in the rate of upper-limb swelling on the operative side between the two groups. The main reason might be that the pressure at the radial artery puncture point can be set according to the individual SBP in the experimental group. Moreover, the compression time is also reduced, decreasing the rate and degree of swelling.

In the present study, the intervention group had a lower incidence of DPI but a similar rate of rebleeding compared with the control group. It might be because a more uniform pressure was distributed on the surface and the pressure on the puncture site decreased with time in the experimental group.

Nowadays, pain is regarded as the fifth vital sign. Since the pressure was visible and controllable in the experimental group and after 2 h, the compression pressure decreased gradually; the patients felt less pain than those in conventional compression.

The total duration of conventional compression can exceed 4–6 h in clinical practice [21–24]. The occurrence of RAO is about 12% after 6 h vs. 5.5% after 2 h [22]. RAO incidence is not rare in clinical practice, but no RAO occurred in the present study. One reason might be the small sample size of the present study, while the short follow-up period might also contribute to this result. Nonetheless, as mentioned above, compression time is one of the most important factors for RAO. Regarding the decreased compression time with our novel artery compression device, it is reasonable to believe that the rate of RAO would be largely reduced in larger clinical trials.

In addition to its effectiveness and safety, this novel automatic pressure-controlled radial compression device could reduce the workload of medical staff and provide economic benefits.

There are several limitations in the present study. First, as mentioned above, the sample size was relatively small, and the involved patients were from a single hospital; therefore, the results might not be generalizable to other centers. Second, as only a few patients underwent PCI, it remains unclear whether this novel device would have the same effectiveness with the increased dose of heparin. Third, the puncture site might differ for different individuals, and the operator's proficiency might also influence the interpretation of the results. Finally, the results of this pilot study will have to be verified in multicenter, larger-scale trials.

## 5. Conclusion

In conclusion, radial compression time after an invasive coronary procedure is important for reducing TRA-related complications. The novel automatic pressure-controlled radial compression device can dramatically reduce the compressing time and decrease complications associated with conventional devices. Larger clinical trials are needed to verify the efficiency and safety of this novel technique.

## Figures and Tables

**Table 1:** Operation process of the controlled pressure automatic decompression radial artery device.

It applies to all patients regardless of antithrombotic therapy
(1) Withdraw the arterial sheath 2-3 cm, and cover the puncture point with sterile gauze
(2) Press the silicone cushion of the controlled pressure automatic decompression radial artery oppressor vertically above the puncture point
(3) Place the compressor self-adhesive elastic strap with proper tightness on the patient's wrist, and then open the switch to the pressure; the motor will drive the oppression parts clockwise and in screw rotation, slowly raising pressure parts; an applied pressure is transferred through the silica gel cushion to the pressure on the sterile gauze. Quickly remove the arterial sheath when the pressure reaches 200 mmHg
(4) When the pressure reaches 220 mmHg, the compressor stops pressurizing
(5) Observe the area for 3–5 min to make sure there is no oozing of blood
(6) The operator instructs the patients on health-related matters requiring special attention:
General matters needing attention
Severe limb movement is prohibited during transport
When the patient arrives at the ward, the nurses place him in a comfortable position, perform monitoring of blood pressure and blood oxygen saturation of the thumb, and then reduce the pressure of the compressor to the patient's systolic blood pressure value of 10–20 mmHg and ensure that the blood oxygen saturation ≥95%
The compressor will automatically decompress at 30 mmHg/h from the second hour
The patient can call the medical staff at any time when a wrist puncture point to bleeding; the medical staff will give a comprehensive evaluation of the correct treatment
When the decompression value is close to the patient's systolic pressure, an alarm will automatically go off. Our medical staff will observe and remove the compressor after there is no bleeding

**Table 2:** Baseline clinical characteristics.

Variables		Intervention group (*n* = 42)	Control group (*n* = 42)	*p* value
Gender	Male	31	27	0.345
	Female	11	15	

Age (years)		65.24 ± 8.96	65.36 ± 8.82	0.951

Comorbidity	Diabetes	2	1	0.168
	Hypertension	24	25	
	Hyperlipidemia	4	1	
	Two of the above	0	3	
	None	12	12	

Heparin (*U*)		4940.48 ± 938.36	5285.71 ± 918.26	0.092

Heart stent	None	38	39	0.898
	One	3	2	
	Two	1	1	

APTT		36.74 ± 3.69	36.67 ± 3.82	0.938
PT		13.12 ± 2.05	12.79 ± 0.58	0.322
INR		1.03 ± 0.23	0.99 ± 0.07	0.306
Thrombocytes		186.72 ± 45.83	184.38 ± 39.06	0.802

APTT: activated partial thromboplastin time; PT: prothrombin time; INR: international normalized ratio.

**Table 3:** Safety outcomes.

Variables		Intervention group (*n* = 42)	Control group (*n* = 42)	*X* ^2^	*p* value
Rebleeding	Bleeding	3	6	0.498	0.480
	None	39	36		

Upper-limb swelling on the operative side	No swelling	41	6	73.665	*p* < 0.001
	Mild swelling	1	11		
	Moderate swelling	0	10		
	Severe swelling	0	15		

DPI	DPI	0	8	—	0.005
	None	42	34		

RAO	RAO	0	0	—	—
	None	42	42		

*Note.* DPI: device-related pressure injury; RAO: radial artery occlusion.

## Data Availability

The data used to support the findings of this study are available from the corresponding author upon request.
